# The ECHELON-2 Trial: 5-year results of a randomized, phase III study of brentuximab vedotin with chemotherapy for CD30-positive peripheral T-cell lymphoma☆

**DOI:** 10.1016/j.annonc.2021.12.002

**Published:** 2021-12-16

**Authors:** S. Horwitz, O. A. O’Connor, B. Pro, L. Trümper, S. Iyer, R. Advani, N. L. Bartlett, J. H. Christensen, F. Morschhauser, E. Domingo-Domenech, G. Rossi, W. S. Kim, T. Feldman, T. Menne, D. Belada, Á. Illés, K. Tobinai, K. Tsukasaki, S.-P. Yeh, A. Shustov, A. Hüttmann, K. J. Savage, S. Yuen, P. L. Zinzani, H. Miao, V. Bunn, K. Fenton, M. Fanale, M. Puhlmann, T. Illidge

**Affiliations:** 1 Memorial Sloan Kettering Cancer Center, New York; 2 University of Virginia Cancer Center, University of Virginia, Charlottesville; 3 Division of Hematology and Oncology, Department of Medicine, Northwestern University Feinberg School of Medicine, Chicago, USA; 4 Universitätsmedizin Göttingen, Göttingen, Germany; 5 MD Anderson Cancer Center/University of Texas, Houston; 6 Stanford Cancer Center, Blood and Marrow Transplant Program, Stanford; 7 Washington University School of Medicine, St. Louis, USA; 8 Odense University Hospital, Odense, Denmark; 9 CHRU de Lille, Lille cedex, Nord-Pas-de-Calais, France; 10 Institut Catala D’oncologia, L’Hospitalet de Llobregat, Barcelona, Spain; 11 Azienda Ospedaliera Spedali Civili di Brescia, Brescia, Italy; 12 Sungkyunkwan University School of Medicine, Samsung Medical Center, Seoul, Republic of Korea; 13 John Theurer Cancer Center, Hackensack Meridian Health School of Medicine, Hackensack, USA; 14 Freeman Hospital, Newcastle upon Tyne, UK; 15 Fourth Department of Internal Medicine - Haematology, Charles University Hospital and Faculty of Medicine, Hradec Králové, Czech Republic; 16 Debreceni Egyetem, Debrecen, Hajdu-Bihar, Hungary; 17 National Cancer Center Hospital, Tokyo; 18 Saitama Medical University International Medical Center, Saitama, Japan; 19 China Medical University Hospital, Taichung, Taiwan; 20 University of Washington Medical Center, Seattle, USA; 21 Universitatsklinikum Essen, Essen, Nordrhein-Westfalen, Germany; 22 Department of Medical Oncology and University of British Columbia, BC Cancer, Vancouver, Canada; 23 Calvary Mater Newcastle Hospital, Waratah, Australia; 24 IRCCS Azienda Ospedaliero-Universitaria di Bologna, Istituto di Ematologia ‘Seràgnoli’, Bologna; 25 Dipartimento di Medicina Specialistica, Diagnostica e Sperimentale, Università di Bologna, Bologna, Italy; 26 Millennium Pharmaceuticals, Inc., Cambridge, a wholly owned subsidiary of Takeda Pharmaceutical Company Limited; 27 Seagen Inc., Bothell, USA; 28 Division of Cancer Sciences, Faculty of Biology, Medicine and Health, University of Manchester, NIHR Biomedical Research Centre, Manchester Academic Health Sciences Centre, Christie Hospital NHS Foundation Trust, Manchester, UK

**Keywords:** brentuximab vedotin, CHOP, frontline treatment, peripheral T-cell lymphoma, randomized clinical trial, overall survival

## Abstract

**Background:**

For patients with peripheral T-cell lymphoma (PTCL), outcomes using frontline treatment with cyclophosphamide, doxorubicin, vincristine, and prednisone (CHOP) or CHOP-like therapy are typically poor. The ECHELON-2 study demonstrated that brentuximab vedotin plus cyclophosphamide, doxorubicin, and prednisone (A+CHP) exhibited statistically superior progression-free survival (PFS) per independent central review and improvements in overall survival versus CHOP for the frontline treatment of patients with systemic anaplastic large cell lymphoma or other CD30-positive PTCL.

**Patients and methods:**

ECHELON-2 is a double-blind, double-dummy, randomized, placebo-controlled, active-comparator phase III study. We present an exploratory update of the ECHELON-2 study, including an analysis of 5-year PFS per investigator in the intent-to-treat analysis group.

**Results:**

A total of 452 patients were randomized (1 : 1) to six or eight cycles of A+CHP (*N* = 226) or CHOP (*N* = 226). At median follow-up of 47.6 months, 5-year PFS rates were 51.4% [95% confidence interval (CI): 42.8% to 59.4%] with A+CHP versus 43.0% (95% CI: 35.8% to 50.0%) with CHOP (hazard ratio = 0.70; 95% CI: 0.53–0.91), and 5-year overall survival (OS) rates were 70.1% (95% CI: 63.3% to 75.9%) with A+CHP versus 61.0% (95% CI: 54.0% to 67.3%) with CHOP (hazard ratio = 0.72; 95% CI: 0.53–0.99). Both PFS and OS were generally consistent across key subgroups. Peripheral neuropathy was resolved or improved in 72% (84/117) of patients in the A+CHP arm and 78% (97/124) in the CHOP arm. Among patients who relapsed and subsequently received brentuximab vedotin, the objective response rate was 59% with brentuximab vedotin retreatment after A+CHP and 50% with subsequent brentuximab vedotin after CHOP.

**Conclusions:**

In this 5-year update of ECHELON-2, frontline treatment of patients with PTCL with A+CHP continues to provide clinically meaningful improvement in PFS and OS versus CHOP, with a manageable safety profile, including continued resolution or improvement of peripheral neuropathy.

## INTRODUCTION

Peripheral T-cell lymphoma (PTCL) is a heterogeneous malignancy that is relatively rare, accounting for ~10% of all non-Hodgkin lymphomas (NHLs) in Western populations.^1^ The most common subtypes of PTCL are the so-called ‘nodal’ PTCLs: PTCL-not otherwise specified (PTCL-NOS), angioimmunoblastic T-cell lymphoma (AITL), and systemic anaplastic large cell lymphoma (sALCL).^1^ sALCL is subdivided into those that harbor a chromosomal translocation involving the anaplastic lymphoma kinase (*ALK*) gene, called ALK-positive (ALK+) sALCL, and those that do not (ALK-negative, ALK−). Although ALK+ sALCL has more favorable prognosis in younger patients, older patients or those with higher international prognostic index (IPI) scores (≥2) can have a prognosis that is similar to ALK− sALCL.^2^

The rarity of PTCL, the diversity of histologies, and diagnostic challenges have hampered progress in treatment of the disease. Historically, the most common treatment of PTCL has been the combination of cyclophosphamide, doxorubicin, vincristine, and prednisone (CHOP) or CHOP-like regimens.^1,3^ Outcomes with CHOP or CHOP-like regimens depend on histology, but are typically poor, with ~75% of patients not responding to or eventually relapsing after initial chemotherapy.^4^ Most relapses of PTCL are in the first 1 to 2 years, but later relapses do occur. Data from the International T-cell Lymphoma Project demonstrated that 37% of relapses post frontline therapy occur after 12 months.^5^ A large international cohort study found that only 36% of PTCL patients were event-free at 24 months, and for these patients, the risk of relapse in the subsequent 5 years was 23%.^6^ For patients who relapse after or are refractory to primary therapy, outcomes are poor and survival is short.^7^ Prior attempts to intensify treatment to improve efficacy have shown only modest or equivocal improvements and lack definitive evidence from a randomized trial. Historically, attempts have been made to improve treatment generally by adding a novel agent to CHOP, but most have outcomes comparable with CHOP and often have significant toxicity.^8–12^

By diagnosis, CD30 is highly expressed in sALCL, but expression is more variable among patients with other PTCL subtypes.^13^ CD30 expression at any level is detected by immunohistochemistry (IHC) in ~60% of PTCL-NOS cases and about half of all AITL cases.^13^ With encouraging results and safety demonstrated from a phase I study,^14^ the phase III ECHELON-2 (NCT01777152) compared frontline treatment of brentuximab vedotin, an antibody-drug conjugate directed to CD30, plus cyclophosphamide, doxorubicin, and prednisone (A+CHP) versus CHOP for patients with sALCL or other CD30-positive PTCL (CD30 ≥10%).^15^ In the primary analysis (median follow-up 36.2 months), the 3-year progression-free survival (PFS) was 57.1% [95% confidence interval (CI): 49.9% to 63.7%] for A+CHP compared with 44.4% with CHOP (95% CI: 37.6% to 50.9%). OS also favored A+CHP over CHOP, with 3-year OS of 76.8% (95% CI: 70.4% to 82.0%) with A+CHP versus 69.1% for CHOP (95% CI: 62.3% to 74.9%). Brentuximab vedotin was approved in combination with CHP in the USA, Europe, Canada, and other parts of the world either for treatment of patients with previously untreated sALCL or more broadly for treatment of patients with previously untreated CD30-expressing PTCL and has since become an accepted standard-of-care option.^16^

Here, we present an exploratory analysis of the ECHELON-2 study at 5 years, including PFS per investigator and OS in the intent-to-treat (ITT) population, results in prespecified subgroups, response rates with brentuximab vedotin treatment after frontline therapy, and peripheral neuropathy (PN) resolution and improvement.

## METHODS

### Study design and patients

ECHELON-2 is a double-blind, double-dummy, randomized, placebo-controlled, active-comparator phase III study. The study design has been described in detail previously.^15^ Briefly, eligible patients were aged 18 years and over and Eastern Cooperative Oncology Group performance status ≤2 with previously untreated CD30-positive PTCL (CD30 detected in ≥10% of neoplastic cells by local review. In cases where enumeration of neoplastic cells was not possible, total lymphocytes were used). Eligible histologies included ALK+ sALCL with an IPI score ≥2, ALK− sALCL, PTCL-NOS, AITL, adult T-cell leukemia/lymphoma, enteropathy-associated T-cell lymphoma, and hepatosplenic T-cell lymphoma. Enrollment targeted 75% ± 5% of patients with sALCL to comply with European and Canadian regulatory requirements for this confirmatory trial. The trial was conducted in accordance with regulatory requirements, and the protocol was approved by institutional review boards and ethics committees at individual sites. All patients provided written informed consent.

### Procedures

Randomization and masking have been described previously.^15^ Patients were treated with six or eight 21-day cycles of either brentuximab vedotin, cyclophosphamide, doxorubicin, prednisone (A+CHP) or cyclophosphamide, doxorubicin, vincristine, prednisone (CHOP). Consolidative stem cell transplantation (SCT) (autologous or allogeneic) or radiotherapy was permitted after treatment at the investigator’s discretion, with intent to transplant specified before to the first cycle of treatment. Data on subsequent therapies, including brentuximab vedotin or brentuximab vedotin-containing regimens, were collected.

### Endpoints and assessments

The primary endpoint of PFS was assessed per blinded independent central review in the prespecified primary analysis. This update at 5 years is exploratory as it was not prespecified in the protocol. The analysis was conducted per investigator following the closure of the blinded independent central reader after completion of the primary analysis. PFS per investigator was defined as the time from the date of randomization to the date of first documentation of relapse or progressive disease,^17^ death due to any cause, or receipt of subsequent systemic chemotherapy to treat residual or progressive PTCL as determined by the investigator, whichever came first. Key alpha-controlled secondary endpoints were OS, PFS in sALCL, complete remission (CR) rate, and objective response rate (ORR). In long-term follow-up, computed tomography scans were required at 9, 12, 15, 18, 21, and 24 months after initiation of study treatment, and every 6 months thereafter until the patient experienced disease progression, death, or analysis of the primary endpoint, whichever came first. Classification of SCT in ECHELON-2 distinguished between use as consolidation and use as subsequent anticancer therapy. The patients classified as receiving SCT as subsequent anticancer therapy had either progressive disease or another non-consolidative subsequent therapy before SCT. Response to subsequent therapies for recurrent or progressive disease after frontline therapy was assessed by the investigator. The relationship between CD30 expression (assessed in local laboratories using the IHC Ber H2 antibody) above/below the median expression level and CR rate and ORR in patients with AITL and PTCL-NOS treated with A+CHP was also assessed in an exploratory *post hoc* analysis. Adverse events (AEs) were defined using Medical Dictionary for Regulatory Activities version 21.0 and the National Cancer Institute Common Terminology Criteria for Adverse Events, version 4.03. Resolution and improvement of PN were monitored during extended follow-up. Data collection is now complete and the study is now closed.

### Statistical analyses

The data cut-off date for this analysis was 5 November 2020. PFS was assessed using the Kaplan–Meier method. Stratification factors included histologic subtype (ALK+ sALCL versus all other histologies) and baseline IPI score (0–1 versus 2–3 versus 4–5). Hazard ratios (HRs) (A+CHP/CHOP) and 95% CIs were based on a stratified Cox’s proportional hazard regression model with treatment as the explanatory variable. Efficacy evaluations were carried out in the ITT population unless otherwise specified. *P* values are nominal and not adjusted for multiplicity. Safety was analyzed in those who received any amount of brentuximab vedotin or any component of CHOP (the safety population). Patients who discontinued treatment due to AEs were included in the analyses provided they were in a CR at end of treatment (EOT). Analyses were conducted with SAS version 9.4 (SAS Institute, Cary, NC).

## RESULTS

From 24 January 2013 to 07 November 2016, a total of 452 patients in 17 countries were randomly assigned to receive A+CHP (*N* = 226) or CHOP (*N* = 226) and followed for a median of 66.8 months (range: 0–90 months) to last contact (Supplementary Figure S1, available at https://doi.org/10.1016/j.annonc.2021.12.002). Baseline patient demographics and disease characteristics were balanced between treatment groups as previously described.^15^ Most patients had advanced stage disease: stage III (124/452; 27%) and stage IV (240/452; 53%). The majority of patients (351/452; 78%) had an IPI score of ≥2. As expected, given the target enrollment, most patients [316/452 (70%)] had sALCL, including 218 (48%) ALK− and 98 (22%) ALK+. For patients with ALK+ sALCL, only those with IPI score ≥2 were enrolled. Among patients with ALK− sALCL, 73% (82/113) in the A+CHP arm were in CR at EOT, and 30% (25/82) underwent consolidative SCT. Among patients with histologies other than sALCL, 64% (41/64) in the A+CHP arm were in CR at EOT and 29% (12/41) underwent consolidative SCT.

**Figure 1.**
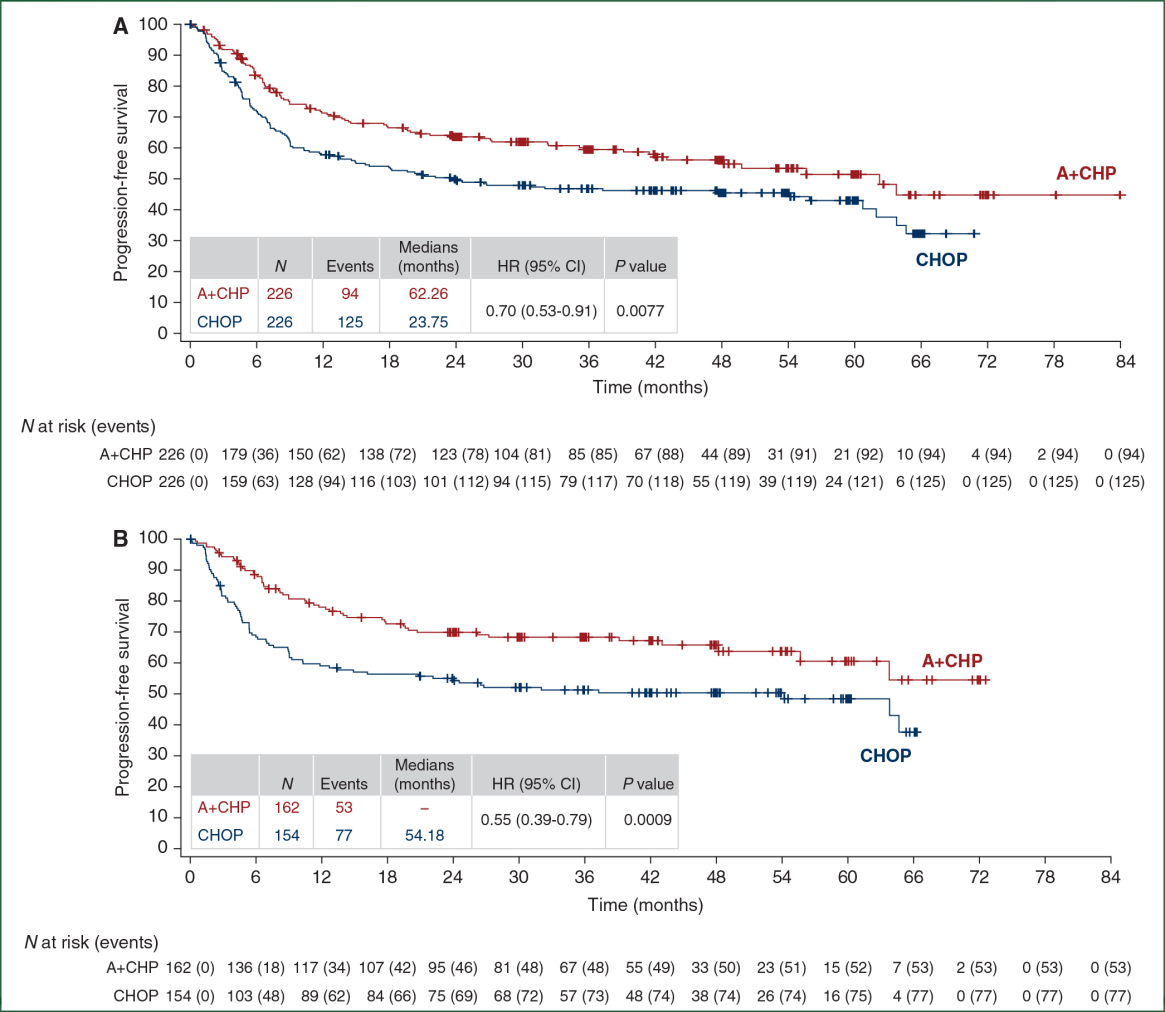
Progression-free survival per investigator by treatment arm. Kaplan–Meier estimates of progression-free survival per investigator by treatment arm for the intent-to-treat population (A) and for patients with systemic anaplastic large cell lymphoma (sALCL) (B). Tick marks indicate censored data. Hazard ratios (A+CHP/CHOP) and 95% CIs were based on a stratified Cox’s proportional hazard regression model with treatment as the explanatory variable. Stratification factors included histologic subtype (ALK+ sALCL versus all other histologies) and baseline international prognostic index (IPI) score (0–1 versus 2–3 versus 4–5). *P* values were calculated using a stratified log-rank test. *P* values are nominal and not adjusted for multiplicity. A+CHP, brentuximab vedotin, cyclophosphamide, doxorubicin, and prednisone; CHOP, cyclophosphamide, doxorubicin, vincristine, and prednisone; CI, confidence interval; HR, hazard ratio.

**Figure 2.**
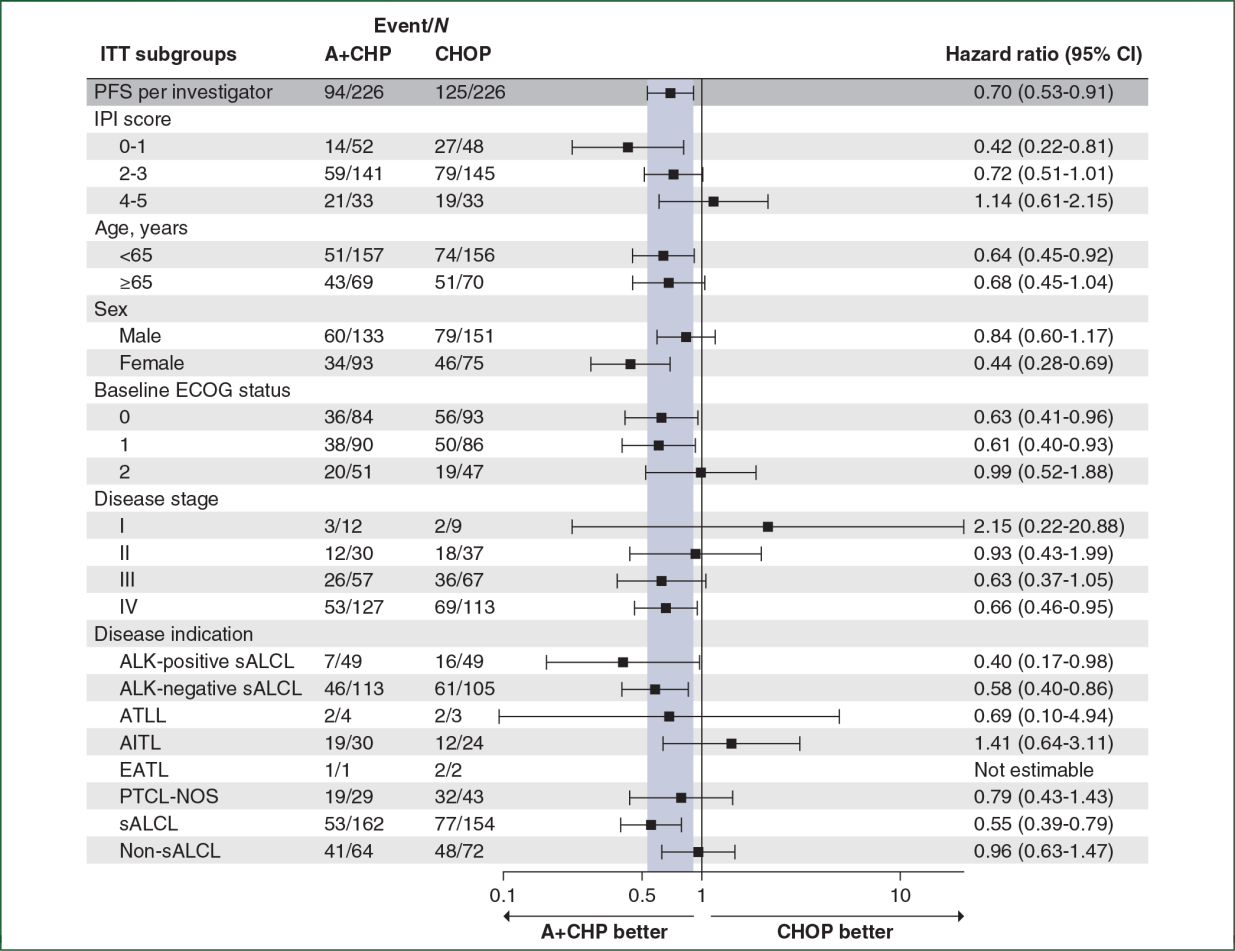
Hazard ratios for progression-free survival per investigator in key prespecified subgroups. This forest plot shows hazard ratios for progression-free survival per investigator in key prespecified subgroups. Hazard ratios are calculated based on a stratified Cox’s proportional hazard regression model considering stratified factors from randomization. A+CHP, brentuximab vedotin, cyclophosphamide, doxorubicin, and prednisone; AITL, angioimmunoblastic T-cell lymphoma; ALK, anaplastic lymphoma kinase; ATLL, adult T-cell leukemia/lymphoma; CHOP, cyclophosphamide, doxorubicin, vincristine, and prednisone; CI, confidence interval; EATL, enteropathy-associated T-cell lymphoma; ECOG, Eastern Cooperative Oncology Group; IPI, international prognostic index; ITT, intent-to-treat; PFS, progression-free survival; PTCL-NOS, peripheral T-cell lymphoma-not otherwise specified; sALCL, systemic anaplastic large cell lymphoma.

The PFS HR was 0.70 (95% CI: 0.53–0.91, *P* = 0.0077), for a 30% reduction in progression events with A+CHP versus CHOP (Figure 1). The median PFS was 62.3 months (95% CI: 42.0 months to not evaluable) for A+CHP and 23.8 months (95% CI: 13.6–60.8 months) for CHOP. The estimated 5-year PFS was 51.4% (95% CI: 42.8% to 59.4%) for the A+CHP arm versus 43.0% (95% CI: 35.8% to 50.0%) for the CHOP arm. The PFS analyses for key prespecified subgroups were generally consistent with the overall study results (Figure 2). The PFS HR was 0.55 (95% CI: 0.39–0.79; *P* = 0.0009) in the subset of patients with sALCL (Figure 1), and the estimated 5-year PFS was 60.6% (95% CI: 49.5% to 69.9%) for A+CHP and 48.4% (95% CI: 39.6% to 56.7%) for CHOP. In patients with AITL, the estimated 5-year PFS was 26.6% (95% CI: 8.9% to 48.4%) for A+CHP and 48.1% (95% CI: 26.6% to 66.8%) for CHOP. In patients with PTCL-NOS, the estimated 5-year PFS was 26.5% (95% CI: 10.9% to 45.2%) for A+CHP and 25.7% (95% CI: 13.1% to 40.4%) for CHOP.

**Figure 3.**
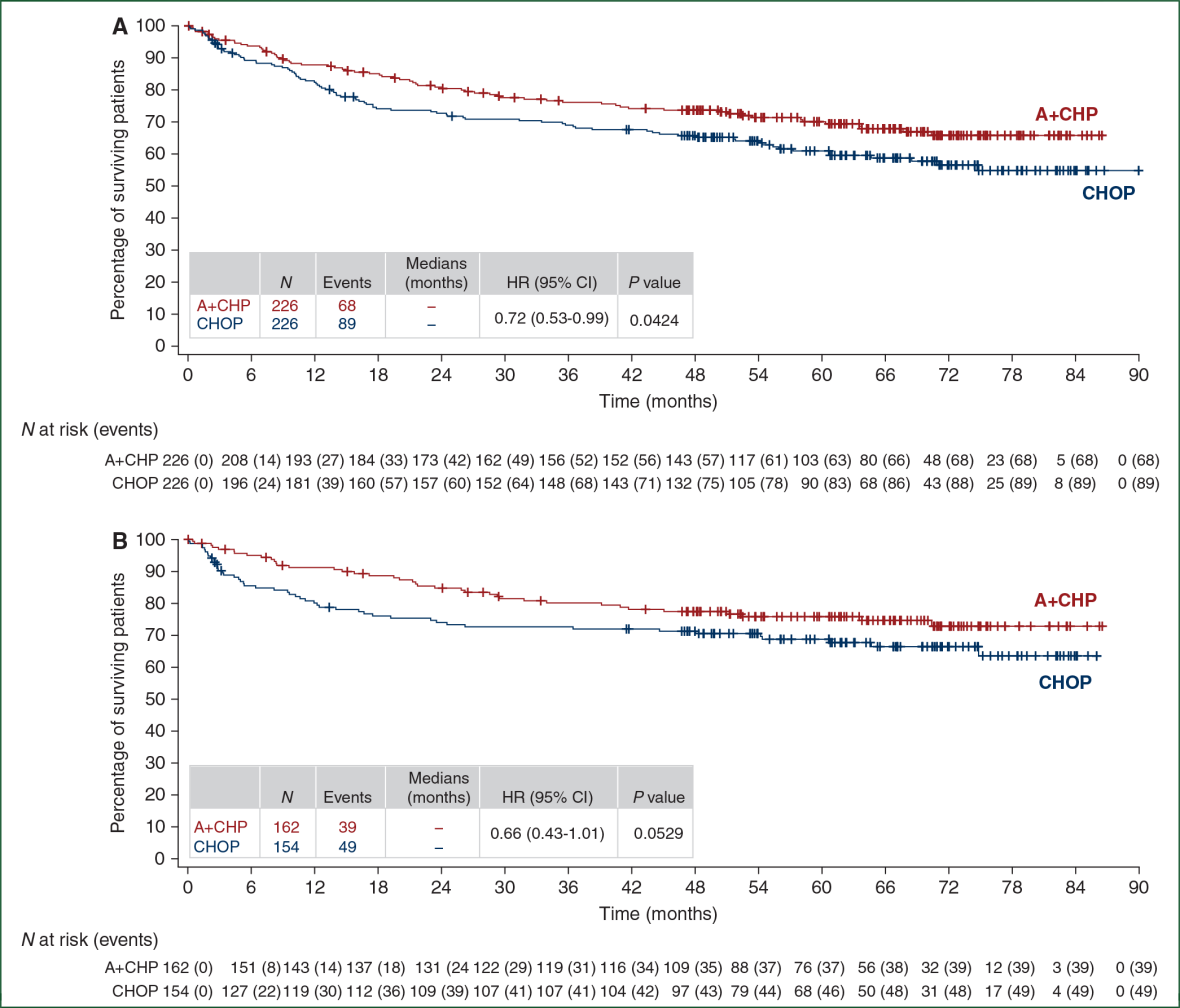
Overall survival by treatment arm. Kaplan-Meier estimates of overall survival by treatment arm for the intent-to-treat population (A) and for patients with sALCL (B). Tick marks indicate censored data. Hazard ratios (A+CHP/CHOP) and 95% CIs were based on a stratified Cox’s proportional hazard regression model with treatment as the explanatory variable. Stratification factors included histologic subtype (ALK+ sALCL versus all other histologies) and baseline IPI score (0–1 versus 2–3 versus 4–5). *P* values were calculated using a stratified log-rank test. *P* values are nominal and not adjusted for multiplicity. A+CHP, brentuximab vedotin, cyclophosphamide, doxorubicin, and prednisone; CHOP, cyclophosphamide, doxorubicin, vincristine, and prednisone; CI, confidence interval; HR, hazard ratio.

**Figure 4.**
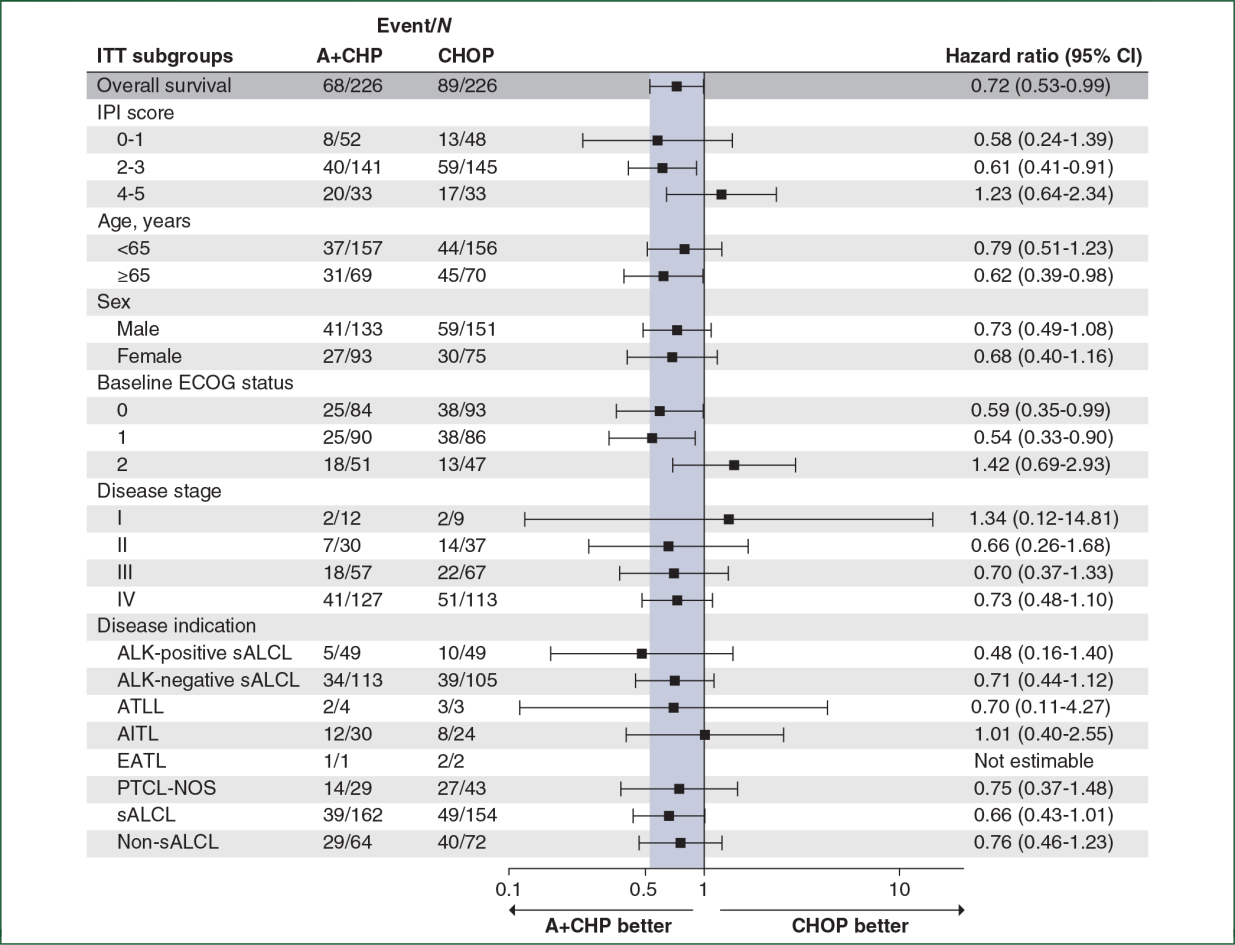
Hazard ratios for overall survival per investigator in key prespecified subgroups. This forest plot shows overall survival in key prespecified subgroups. Hazard ratios are calculated based on a stratified Cox’s proportional hazard regression model considering stratified factors from randomization. A+CHP, brentuximab vedotin, cyclophosphamide, doxorubicin, and prednisone; AITL, angioimmunoblastic T-cell lymphoma; ALK, anaplastic lymphoma kinase; ATLL, adult T-cell leukemia/lymphoma; CHOP, cyclophosphamide, doxorubicin, vincristine, and prednisone; CI, confidence interval; EATL, enteropathy-associated T-cell lymphoma; ECOG, Eastern Cooperative Oncology Group; IPI, international prognostic index; ITT, intent-to-treat; PFS, progression-free survival; PTCL-NOS, peripheral T-cell lymphoma-not otherwise specified; sALCL, systemic anaplastic large cell lymphoma.

The OS HR was 0.72 (95% CI: 0.53–0.99, *P* = 0.0424) (Figure 3). The estimated 5-year OS was 70.1% (95% CI: 63.3% to 75.9%) for the A+CHP arm versus 61.0% (95% CI: 54.0% to 67.3%) for the CHOP arm. Median OS was not reached in either arm. The OS analyses for key prespecified subgroups were generally consistent with the overall study results (Figure 4). In patients with sALCL, the OS HR was 0.66 (95% CI: 0.43–1.01, *P* = 0.0529) (Figure 3), and 5-year OS was 75.8% (95% CI: 68.2% to 81.9%) for the A+CHP arm versus 68.7% (95% CI: 60.5% to 75.6%) for the CHOP arm. In patients with AITL, the estimated 5-year OS was 67.8% (95% CI: 46.8% to 81.9%) for A+CHP and 62.5% (95% CI: 37.7% to 79.7%) for CHOP. In patients with PTCL-NOS, the estimated 5-year OS was 46.2% (95% CI: 26.1% to 64.2%) for A+CHP and 35.9% (21.0% to 51.1%) for CHOP.

Most (26/29, 90%) AITL patients had CD30 expression between 10% and 30%; CD30 expression among patients with PTCL-NOS was more widely distributed from 10% to 100% (Supplementary Figure S2A, available at https://doi.org/10.1016/j.annonc.2021.12.002). Median CD30 expression was 18% in AITL and 25% in PTCL-NOS. Level of CD30 expression, when assessed as above or below the median for the subtype, found no apparent correlation with likelihood of CR for patients with either AITL or PTCL-NOS (Supplementary Table S1, available at https://doi.org/10.1016/j.annonc.2021.12.002) and responses were observed across the range of CD30 expression (Supplementary Figure S2B and C, available at https://doi.org/10.1016/j.annonc.2021.12.002).

Use of SCT as consolidation was reported for 50 (22%) patients in the A+CHP arm and 39 (17%) in the CHOP arm (Supplementary Table S2, available at https://doi.org/10.1016/j.annonc.2021.12.002). Autologous SCT as consolidation was reported in 49 (22%) patients and 39 (17%) patients in the A+CHP and CHOP arms, respectively.

Subsequent anticancer therapies were reviewed; 70 (31%) of 226 patients in the A+CHP arm and 102 (45%) of 226 patients in the CHOP arm received subsequent anticancer therapy. Nearly all subsequent therapy was received as systemic treatment of residual/progressive disease (Supplementary Table S3, available at https://doi.org/10.1016/j.annonc.2021.12.002). In total, >20 different treatments or regimens were employed for patients with progressive and/or relapsed disease (Supplementary Table S3, available at https://doi.org/10.1016/j.annonc.2021.12.002). SCT used as subsequent anticancer therapy was reported for 23 (10%) patients in the A+CHP arm and 31 (14%) patients in the CHOP arm [autologous in 14 (6%) patients in the A+CHP arm and 13 (6%) patients in the CHOP arm]. Not including SCT, the most common subsequent therapies in the A+CHP and CHOP arms were brentuximab vedotin monotherapy [24 patients (11%) and 47 patients (21%), respectively] and platinum-based combination chemotherapy [21 patients (9%) and 42 patients (19%), respectively]. The median number of types of subsequent anticancer therapies received per patient was 1 (range, 1–3) in both treatment arms. The ORR with first subsequent therapy on the A+CHP arm was 42% (27% CR) versus 42% (19% CR) in the CHOP arm.

A total of 29 of 226 patients (13%) in the A+CHP arm (19 sALCL, 5 PTCL-NOS, and 5 AITL) and 54 of 226 patients (24%) in the CHOP arm (39 sALCL, 10 PTCL-NOS, 4 AITL, and 1 EATL) received systemic therapy with brentuximab vedotin after experiencing progressive disease or relapse after frontline therapy (Table 1; Supplementary Table S2, available at https://doi.org/10.1016/j.annonc.2021.12.002). In the A+CHP arm, the median time to brentuximab vedotin retreatment [as monotherapy (*N* = 25) or combination therapy (*N* = 4)] after frontline therapy was 15.0 months (range, 3–64 months), and the median treatment duration was 2.1 months (range, 0–18 months); 17 patients (ORR: 59%) had CR (*N* = 11) or partial remission (PR) (*N* = 6) after retreatment by investigator assessment. In the CHOP arm, the median time to brentuximab vedotin treatment monotherapy (*N* = 48) or combination therapy (*N* = 6) after frontline CHOP therapy was 8.2 months (range, 1–67 months) and the median duration was 2.2 months (range, 0–11 months); 27 patients (ORR: 50%) had CR (*N* = 16) or PR (*N* = 11).

PN events were reported in 117 (52%) patients in the A+CHP arm and 124 (55%) patients in the CHOP arm.^15^ At last follow-up, 47 (21%) patients in the A+CHP had ongoing PN (33, 13, and 1 patient with grade 1, 2, and 3, respectively). In the CHOP arm, 42 (19%) patients had ongoing PN (30, 11, and 1 patient with grade 1, 2, and 3, respectively). Most events of PN had improved or resolved in both arms, including 72% of patients (84 of 117) with PN in the A+CHP arm and 78% of patients (97 of 124) in the CHOP arm. In the A+CHP arm, maximum severity was grade 1 for 33 (70%) of 47 patients, grade 2 for 13 (28%), and grade 3 for 1 (2%). In the CHOP arm, maximum severity was grade 1 for 30 (64%) of 42 patients, grade 2 for 11 (23%), and grade 3 for 1 (2%). Assessment of PN outcome of grade 2 or 3 events was confounded in 19 of 29 patients by death (*N* = 12), withdrawal from the study (*N* = 6), and loss to follow-up (*N* = 1).

Secondary malignancies were reported in 14 patients: 6 in the A+CHP arm and 8 in the CHOP arm. In the A+CHP arm, the malignancies included three hematological malignancies (including one case of myelodysplastic syndrome), two solid tumors, and one malignancy without information on type. In the CHOP arm, six hematological and two solid tumors occurred, including one case of myelodysplastic syndrome. Use of subsequent anticancer therapy, including autologous SCT, was not reported for any of the patients with a secondary malignancy.

## DISCUSSION

With 5 years of follow-up, the ECHELON-2 study continues to demonstrate that frontline treatment of patients with PTCL with A+CHP provides a clinically meaningful improvement in OS over CHOP. Treatment with A+CHP also maintains its survival benefit over CHOP, with a 5-year OS of 70.1% versus 61.0%, respectively, demonstrating a 28% reduction in the risk of death (HR = 0.72; 95% CI: 0.53–0.99), and thus ECHELON-2 remains the only frontline PTCL trial to demonstrate an OS benefit.

The safety profile remains manageable and very similar to CHOP, including no increase in secondary malignancies. PN events continue to resolve and improve, and the proportion of patients with ongoing PN is similar in the two treatment arms.

Significant improvement in treatment approaches for PTCL has been hampered by the rarity and heterogeneity of the disease; data from large, prospective, randomized studies have not been available to inform treatment guidelines. Studies testing agents to improve upon the efficacy of CHOP, CHOP with an added novel agent, or alternative combination chemotherapy regimens have largely been single-arm or phase II studies, with little evidence of improved efficacy and often with increased toxicity.^16^ Prospective, randomized studies of cyclophosphamide, doxorubicin, vincristine, etoposide, and prednisone (CHOEP) versus CHOP have been carried out primarily for aggressive B-cell lymphoma (NHL-B1 and NHL-B2), with patients with PTCL making up <10% of the enrolled subjects.^18,19^ Although there is evidence of clinical benefit in retrospective analyses, particularly in patients aged 60 years and younger, no OS benefit has been demonstrated, and CHOEP was associated with increased toxicity, particularly myelosuppression, in those more than 60 years of age.^20^ Challenges in improving outcomes for patients with PTCL, including with novel agents, was further highlighted by the recent failure of a large randomized study of romidepsin plus CHOP: no significant improvement in response, PFS, or OS, and an increase in hematologic toxicities were reported.^21^

ECHELON-2 is the largest prospective, randomized frontline trial ever conducted for patients with PTCL. The study met its primary endpoint, establishing A+CHP as an accepted standard-of-care option for CD30-positive PTCLs,^16^ and this 5-year analysis confirms continued benefit of A+CHP over CHOP. The study enrolled a high number of sALCL patients to comply with EU and Canadian regulatory guidance and was not powered to compare A+CHP versus CHOP among non-ALCL histologic subtypes. Furthermore, this 5-year analysis of ECHELON-2 provides a valuable and unique dataset benchmarking long-term outcomes of patients treated with standard CHOP, who in this study achieved a higher PFS and OS than previous retrospective datasets.^1,4^ Notably, patients more than 65 years of age had a significant improvement in outcome with A+CHP in ECHELON-2 (Figures 2 and 4). Although growth factor support was not mandated in this study, recommendations are for primary prophylaxis in all patients.

ECHELON-2 used a CD30 cut-off of 10% for patient eligibility and thus does not provide information on efficacy of A+CHP in patients with no or low CD30 expression. Notably, regulatory approvals for brentuximab vedotin do not include a minimum threshold for CD30 expression. In patients with non-ALCL histologies in ECHELON-2, there was no apparent correlation between CD30 expression levels and the likelihood of response (Supplementary Table S1, Supplementary Figure S2, available at https://doi.org/10.1016/j.annonc.2021.12.002). Although this exploratory *post hoc* analysis was not powered to detect the effect of CD30 expression on the likelihood of response, these results further support prior observations that clinical benefit from brentuximab vedotin can occur at all levels of CD30 expression.^22^ This may be attributed to postulated CD30-independent mechanisms of action, including antibody-dependent cellular phagocytosis, immunogenic cell death, the bystander effect, and depletion of CD30-positive T regulatory cells.^23–25^ In a phase II study, CR or PR was reported in 4 of 12 patients with relapsed PTCL with CD30 expression <10%.^26,27^ Similar results have been observed in CTCL^28,29^ and B-cell lymphomas.^30,31^ These clinical data, in conjunction with nonclinical studies,^24,25,32^ support the prospective evaluation of A+CHP in patients with non-sALCL PTCL and CD30 expression <10% on tumor cells, and a phase II study has been initiated (NCT04569032).^33^

The heterogeneity among subsequent therapies observed in this trial is notable and consistent with the wide range of accepted therapeutic approaches as well as the remaining high therapeutic needs and lack of uniform approaches for patients with aggressive refractory/relapsed PTCL. The ORR with brentuximab vedotin retreatment in ECHELON-2 (59%) is consistent with a small earlier study that evaluated retreatment with brentuximab vedotin in patients who relapsed after achieving a CR or PR.^34^ Although the patient numbers were small, response was assessed by the investigator, and the patients had not received brentuximab vedotin as frontline treatment. The results, in combination with results from ECHELON-2, suggest that retreatment with brentuximab vedotin is a viable option. An ongoing phase II trial is being conducted to provide additional data on retreatment with brentuximab vedotin for patients with classical Hodgkin lymphoma, sALCL, or other CD30-positive PTCL (NCT03947255).^35^

The ECHELON-2 study demonstrated the clinical benefit of adding brentuximab vedotin to CHOP-based chemotherapy for the frontline treatment of patients with CD30-positive PTCL, but further research is being conducted to build upon this therapeutic advance. A phase II trial is ongoing to evaluate the safety and efficacy of the addition of brentuximab vedotin to CHOEP-based chemotherapy (brentuximab vedotin plus cyclophosphamide, doxorubicin, etoposide, and prednisone; A+CHEP) followed by brentuximab vedotin consolidation in patients with newly diagnosed CD30-positive PTCL.^36^ The study population is primarily patients with non-ALCL PTCL, including 38% patients with AITL.^37^ Early data from this trial have been promising. After completion of A+CHEP (*N* = 46), the ORR was 91% (37 CR, 5 PR, 4 progressive disease). A+CHEP resulted in high rates of hematologic toxicity but was reported to be otherwise tolerable; PN was reported in 67%.

In this 5-year update for the ECHELON-2 study, frontline treatment with A+CHP continues to provide clinically meaningful improvement in PFS and OS versus CHOP for patients with PTCL, including ongoing remission in >60% of patients with sALCL at 5 years, with a manageable safety profile, including continued resolution or improvement of PN.

## Supplementary Material

1

2

3

4

5

## Figures and Tables

**Table 1.** Response to first brentuximab vedotin treatment after frontline therapy

	Overall	sALCL	PTCL-NOS	AITL	EATL

A+CHP					
*N*	29	19	5	5	0
Objective response rate, *n* (%)	17 (59)	12 (63)	3 (60)	2 (40)	NA
Complete remission, *n* (%)^a^	11 (38)	8 (42)	2 (40)	1 (20)	NA
Partial remission, *n* (%)^a^	6(21)	4(21)	1 (20)	1 (20)	NA
CHOP					
*N*	54	39	10	4	1
Objective response rate, *n* (%)	27 (50)	23 (59)	3 (30)	1 (25)	0 (0)
Complete remission, *n* (%)^a^	16 (30)	12 (31)	3 (30)	1 (25)	0
Partial remission, *n* (%)^a^	11 (20)	11 (28)	0	0	0

A+CHP, brentuximab vedotin, cyclophosphamide, doxorubicin, and prednisone; AITL, angioimmunoblastic T-cell lymphoma; CHOP, cyclophosphamide, doxorubicin, vincristine, and prednisone; EATL, enteropathy-associated T-cell lymphoma; NA, not applicable; PTCL-NOS, peripheral T-cell lymphoma-not otherwise specified; sALCL, systemic anaplastic large cell lymphoma.

a Responses were assessed by investigators based on Revised Response Criteria for Malignant Lymphoma.^17^ Responses are mutually exclusive.

## Data Availability

De-identified patient-level trial data that underlie the results reported in this publication will be made available upon study completion on a case-by-case basis to researchers who provide a methodologically sound proposal. Additional documentation may also be made available. Data availability will begin after approval of the qualified request and end 30 days after receipt of datasets. All requests can be submitted to CTDR@seagen.com and will be reviewed by an internal review committee. Please note that the data sharing policy of this clinical study’s sponsor, Seagen Inc., requires all requests for clinical trial data be reviewed to determine the qualification of the specific request. This policy is available at https://www.seagen.com/healthcare-professionals/clinical-data-requests and is aligned with BIO’s Principles on Clinical Trial Data Sharing (available at https://www.bio.org/blogs/principles-clinical-trial-data-sharing-reaffirm-commitment).

## References

[1] Vose J, Armitage J, Weisenburger D, International T-Cell lymphoma project. International peripheral T-cell and natural killer/T-cell lymphoma study: pathology findings and clinical outcomes. J Clin Oncol. 2008;26:4124–4130. 18626005 10.1200/JCO.2008.16.4558

[2] Savage KJ, Harris NL, Vose JM, ALK- anaplastic large-cell lymphoma is clinically and immunophenotypically different from both ALK+ ALCL and peripheral T-cell lymphoma, not otherwise specified: report from the International Peripheral T-Cell Lymphoma Project. Blood. 2008;111:5496–5504. 18385450 10.1182/blood-2008-01-134270

[3] Chihara D, Fanale MA, Miranda RN, The survival outcome of patients with relapsed/refractory peripheral T-cell lymphoma-not otherwise specified and angioimmunoblastic T-cell lymphoma. Br J Haematol. 2017;176:750–778. 27983760 10.1111/bjh.14477 PMC5836501

[4] Ellin F, Landstrom J, Jerkeman M, Relander T. Real-world data on prognostic factors and treatment in peripheral T-cell lymphomas: a study from the Swedish Lymphoma Registry. Blood. 2014;124:1570–1577. 25006130 10.1182/blood-2014-04-573089

[5] Bellei M, Foss FM, Shustov AR, The outcome of peripheral T-cell lymphoma patients failing first-line therapy: a report from the prospective, International T-cell Project. Haematologica. 2018;103:1191–1197. 29599200 10.3324/haematol.2017.186577 PMC6029527

[6] Maurer MJ, Ellin F, Srour L, International assessment of event-free survival at 24 months and subsequent survival in peripheral T-cell lymphoma. J Clin Oncol. 2017;35:4019–4026. 29072976 10.1200/JCO.2017.73.8195 PMC5736237

[7] Mak V, Hamm J, Chhanabhai M, Survival of patients with peripheral T-cell lymphoma after first relapse or progression: spectrum of disease and rare long-term survivors. J Clin Oncol. 2013;31:1970–1976. 23610113 10.1200/JCO.2012.44.7524

[8] Wulf GG, Altmann B, Ziepert M, Alemtuzumab plus CHOP versus CHOP in elderly patients with peripheral T-cell lymphoma: the DSHNHL2006–1B/ACT-2 trial. Leukemia. 2021;35:143–155. 32382083 10.1038/s41375-020-0838-5

[9] Kluin-Nelemans HC, van Marwijk Kooy M, Lugtenburg PJ, Intensified alemtuzumab-CHOP therapy for peripheral T-cell lymphoma. Ann Oncol. 2011;22:1595–1600. 21212158 10.1093/annonc/mdq635

[10] Foss FM, Sjak-Shie N, Goy A, A multicenter phase II trial to determine the safety and efficacy of combination therapy with denileukin diftitox and cyclophosphamide, doxorubicin, vincristine and prednisone in untreated peripheral T-cell lymphoma: the CONCEPT study. Leuk Lymphoma. 2013;54:1373–1379. 23278639 10.3109/10428194.2012.742521

[11] Deng S, Lin S, Shen J, Zeng Y. Comparison of CHOP vs CHOPE for treatment of peripheral T-cell lymphoma: a meta-analysis. Onco Targets Ther. 2019;12:2335–2342. 30992670 10.2147/OTT.S189825 PMC6445243

[12] Simon A, Peoch M, Casassus P, Upfront VIP-reinforced-ABVD (VIP-rABVD) is not superior to CHOP/21 in newly diagnosed peripheral T cell lymphoma. Results of the randomized phase III trial GOELAMS-LTP95. Br J Haematol. 2010;151:159–166. 20738307 10.1111/j.1365-2141.2010.08329.x

[13] Sabattini E, Pizzi M, Tabanelli V, CD30 expression in peripheral T-cell lymphomas. Haematologica. 2013;98:e81–e82. 23716537 10.3324/haematol.2013.084913 PMC3729886

[14] Fanale MA, Horwitz SM, Forero-Torres A, Five-year outcomes for frontline brentuximab vedotin with CHP for CD30-expressing peripheral T-cell lymphomas. Blood. 2018;131:2120–2124. 29507077 10.1182/blood-2017-12-821009 PMC5946765

[15] Horwitz S, O’Connor OA, Pro B, Brentuximab vedotin with chemotherapy for CD30-positive peripheral T-cell lymphoma (ECHELON-2): a global, double-blind, randomised, phase 3 trial. Lancet. 2019;393:229–240. 30522922 10.1016/S0140-6736(18)32984-2 PMC6436818

[16] Zain JM. Aggressive T-cell lymphomas: 2019 updates on diagnosis, risk stratification and management. Am J Hematol. 2019;94:929–946. 31119775 10.1002/ajh.25513

[17] Cheson BD, Pfistner B, Juweid ME, Revised response criteria for malignant lymphoma. J Clin Oncol. 2007;25:579–586. 17242396 10.1200/JCO.2006.09.2403

[18] Pfreundschuh M, Trumper L, Kloess M, Two-weekly or 3-weekly CHOP chemotherapy with or without etoposide for the treatment of elderly patients with aggressive lymphomas: results of the NHL-B2 trial of the DSHNHL. Blood. 2004;104:634–641. 15016643 10.1182/blood-2003-06-2095

[19] Pfreundschuh M, Trumper L, Kloess M, Two-weekly or 3-weekly CHOP chemotherapy with or without etoposide for the treatment of young patients with good-prognosis (normal LDH) aggressive lymphomas: results of the NHL-B1 trial of the DSHNHL. Blood. 2004;104:626–633. 14982884 10.1182/blood-2003-06-2094

[20] Schmitz N, Trumper L, Ziepert M, Treatment and prognosis of mature T-cell and NK-cell lymphoma: an analysis of patients with T-cell lymphoma treated in studies of the German High-Grade Non-Hodgkin Lymphoma Study Group. Blood. 2010;116:3418–3425. 20660290 10.1182/blood-2010-02-270785

[21] Bachy E, Camus V, Thieblemont C, Romidepsin plus CHOP versus CHOP in patients with previously untreated peripheral T-cell lymphoma: results of the Ro-CHOP phase III study (conducted by LYSA). J Clin Oncol. 2022;40(3):242–251. 34843406 10.1200/JCO.21.01815

[22] Jagadeesh D, Horwitz SM, Bartlett NL, Response to brentuximab vedotin by CD30 expression: results from five trials in PTCL, CTCL, and B-cell lymphomas. J Clin Oncol. 2019;37:7543.

[23] Gardai SJ, Epp A, Law CL. Brentuximab vedotin-mediated immunogenic cell death. Cancer Res. 2015;75:2469.

[24] Li F, Emmerton KK, Jonas M, Intracellular released payload influences potency and bystander-killing effects of antibody-drug conjugates in preclinical models. Cancer Res. 2016;76:2710–2719. 26921341 10.1158/0008-5472.CAN-15-1795

[25] Muller P, Martin K, Theurich S, Microtubule-depolymerizing agents used in antibody-drug conjugates induce antitumor immunity by stimulation of dendritic cells. Cancer Immunol Res. 2014;2:741–755. 24916470 10.1158/2326-6066.CIR-13-0198

[26] Jagadeesh D, Horwitz S, Bartlett NL, Response to brentuximab vedotin by CD30 expression: results from five trials in PTCL, CTCL, and B-cell lymphomas. Hematol Oncol. 2019;37:470–471.

[27] Horwitz SM, Advani RH, Bartlett NL, Objective responses in relapsed T-cell lymphomas with single-agent brentuximab vedotin. Blood. 2014;123:3095–3100. 24652992 10.1182/blood-2013-12-542142 PMC4425442

[28] Prince HM, Kim YH, Horwitz SM, Brentuximab vedotin or physician’s choice in CD30-positive cutaneous T-cell lymphoma (ALCANZA): an international, open-label, randomised, phase 3, multicentre trial. Lancet. 2017;390:555–566. 28600132 10.1016/S0140-6736(17)31266-7

[29] Duvic M, Tetzlaff MT, Gangar P, Clos AL, Sui D, Talpur R. Results of a phase II trial of brentuximab vedotin for CD30+ cutaneous t-cell lymphoma and lymphomatoid papulosis. J Clin Oncol. 2015;33:3759–3765. 26261247 10.1200/JCO.2014.60.3787 PMC4737859

[30] Bartlett NL, Smith MR, Siddiqi T, Brentuximab vedotin activity in diffuse large B-cell lymphoma with CD30 undetectable by visual assessment of conventional immunohistochemistry. Leuk Lymphoma. 2017;58:1607–1616. 27868471 10.1080/10428194.2016.1256481

[31] Jacobsen ED, Sharman JP, Oki Y, Brentuximab vedotin demonstrates objective responses in a phase 2 study of relapsed/refractory DLBCL with variable CD30 expression. Blood. 2015;125:1394–1402. 25573987 10.1182/blood-2014-09-598763

[32] Oflazoglu E, Stone IJ, Gordon KA, Macrophages contribute to the antitumor activity of the anti-CD30 antibody SGN-30. Blood. 2007;110: 4370–4372. 17909075 10.1182/blood-2007-06-097014

[33] Jagadeesh D, Sims RB, Horwitz SM. Trial-in-progress: frontline brentuximab vedotin and CHP (A+CHP) in patients with peripheral T-cell lymphoma with less than 10% CD30 expression. Blood. 2020;136:30.

[34] Bartlett NL, Chen R, Fanale MA, Retreatment with brentuximab vedotin in patients with CD30-positive hematologic malignancies. J Hematol Oncol. 2014;7:24. 24642247 10.1186/1756-8722-7-24 PMC3994656

[35] Ahmed S, Lisano J, Newhook T. A phase 2 study of retreatment with brentuximab vedotin in patients with cHL, sALCL or other CD30-expressing PTCL. Blood. 2019;134:4054.

[36] Herrera AF, Zain J, Savage KJ, Preliminary results from a phase 2 trial of brentuximab vedotin plus cyclophosphamide, doxorubicin, etoposide, and prednisone (CHEP-BV) followed by BV consolidation in patients with CD30-positive peripheral T-cell lymphomas. Blood. 2019;134:4023. 10.1016/S2352-3026(24)00171-6 39067464

[37] Herrera AF, Zain J, Savage KJ, Brentuximab vedotin plus cyclophosphamide, doxorubicin, etoposide, and prednisone (CHEP-BV) followed by BV consolidation in patients with CD30-expressing peripheral T-cell lymphomas. Blood. 2021;138:133.

