# Supplementary material for: The ECHELON-2 Trial: 5-year results of a randomized, phase III study of brentuximab vedotin with chemotherapy for CD30-positive peripheral T-cell lymphoma
Source: Ann Oncol. Author manuscript; Available in PMC 2022 Sep 6. (PMC9447792; doi:10.1016/j.annonc.2021.12.002)

Supplementary Figure S1: CONSORT diagram

*Screening informed consents were obtained for seven subjects to allow sites to perform screening activities that were not considered standard of care at their sites. The remaining 594 subjects signed the full informed consent for the study. †Includes three subjects who were randomized to the A+CHP arm but did not receive study treatment. ‡A total of 89 subjects in the A+CHP arm and 81 subjects in the CHOP arm were prespecified by the investigator at baseline to receive consolidative stem cell transplantation. A+CHP, brentuximab vedotin, cyclophosphamide, doxorubicin, and prednisone; CHOP, cyclophosphamide, doxorubicin, vincristine, and prednisone.


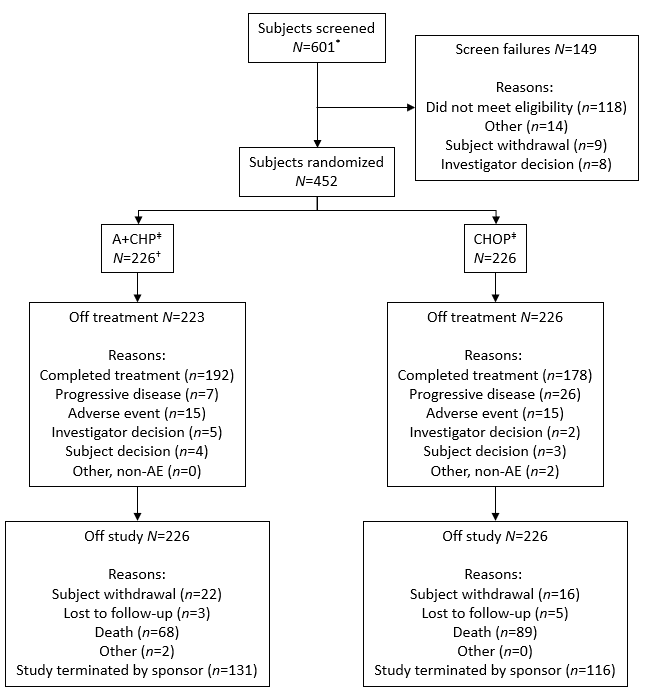

Supplement: 1 [file NIHMS1829921-supplement-1.docx]
