# Supplementary material for: The ECHELON-2 Trial: 5-year results of a randomized, phase III study of brentuximab vedotin with chemotherapy for CD30-positive peripheral T-cell lymphoma
Source: Ann Oncol. Author manuscript; Available in PMC 2022 Sep 6. (PMC9447792; doi:10.1016/j.annonc.2021.12.002)
Supplement: 2 [file NIHMS1829921-supplement-2.docx]

**Supplementary Figure S2: CD30 expression in patients with AITL and PTCL-NOS in the A+CHP treatment arm**

1. CD30 expression in all patients by histology, (B) CD30 expression in patients with AITL by response, and (C) CD30 expression in patients with PTCL by response. CD30 was assessed in local laboratories. EOT, end of treatment; BICR, blinded independent review committee.

**
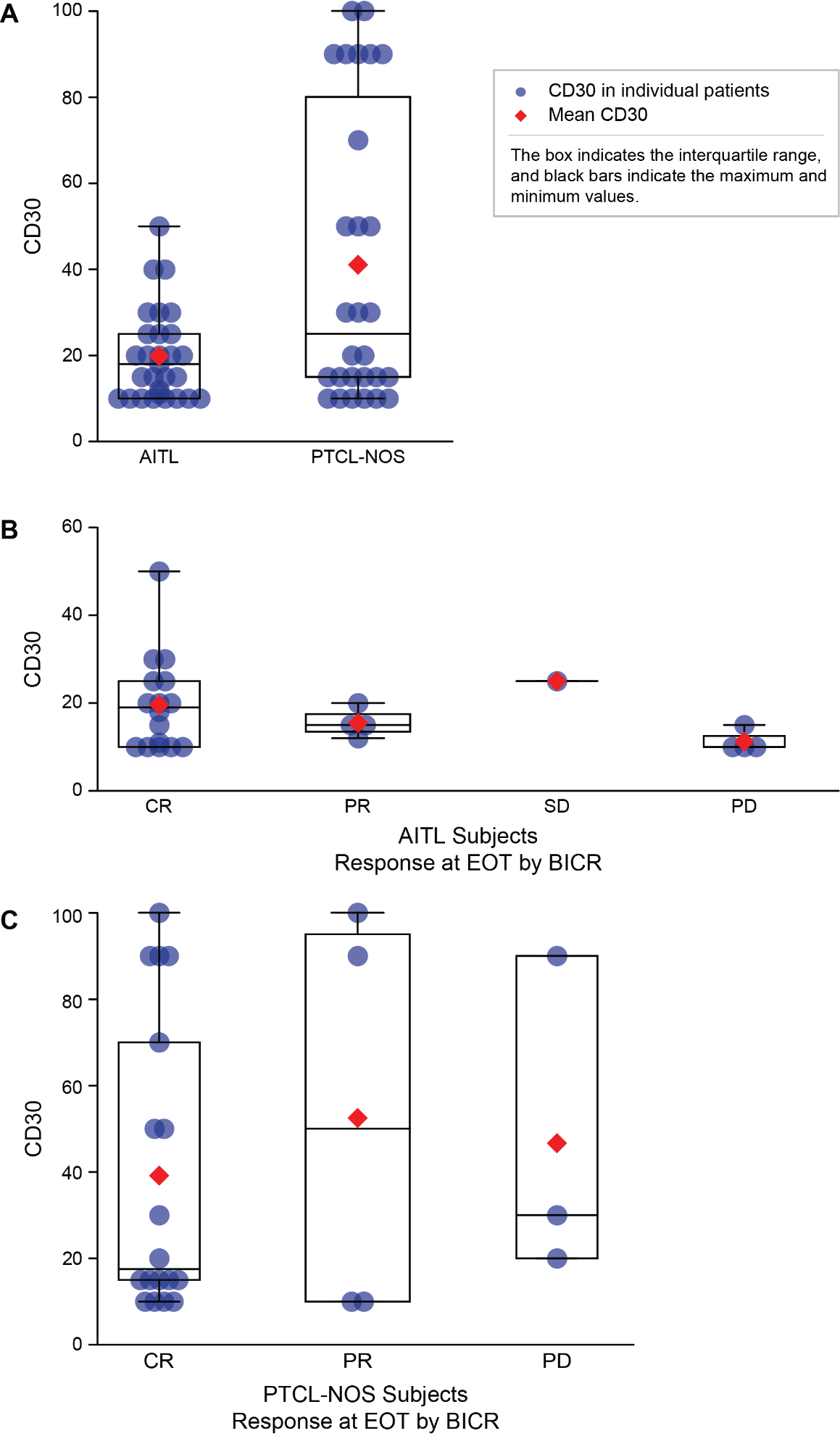
**
