# Supplementary material for: The ECHELON-2 Trial: 5-year results of a randomized, phase III study of brentuximab vedotin with chemotherapy for CD30-positive peripheral T-cell lymphoma
Source: Ann Oncol. Author manuscript; Available in PMC 2022 Sep 6. (PMC9447792; doi:10.1016/j.annonc.2021.12.002)
Supplement: 3 [file NIHMS1829921-supplement-3.docx]

**Supplementary Table S1: CR and PR rates by CD30 expression in patients with AITL or PTCL-NOS in the A+CHP arm**

|  | **CD30** | **Patients *N*** | **Complete remission *n* (%)** | **Partial remission  *n* (%)** | ***P* value, CR rates for CD30 above vs below median ^a^** |
| --- | --- | --- | --- | --- | --- |
| AITL | CD30 > median | 14 | 8 (57) | 1 (7) | 0.84 |
|  | CD30 ≤ median^b^ | 15 | 8 (53) | 3 (20) |  |
|  | CD30 = 10% | 8 | 5 (63) | 0 |  |
| PTCL-NOS | CD30 > median | 14 | 8 (57) | 2 (14) | 0.44 |
|  | CD30 ≤ median^b^ | 14 | 10 (71) | 2 (14) |  |
|  | CD30 = 10% | 6 | 4 (67) | 2 (33) |  |

AITL, angioimmunoblastic T-cell lymphoma; PTCL-NOS, peripheral T-cell lymphoma-not otherwise specified

1. Cochran-Mantel-Haenzel test comparing CR rates in patients with CD30 above vs below median
2. Patients with CD30 = 10% were included in the category CD30 ≤ median

Reprinted from Advani RH, Horwitz SM, Iyer SP, et al. Response to A+CHP by CD30 expression in the ECHELON-2 trial. Poster presented at: Annual Meeting of the American Society of Clinical Oncology; Chicago, IL, May 31-Jun 4, 2019 with permission from the Author.
