# Supplementary material for: The ECHELON-2 Trial: 5-year results of a randomized, phase III study of brentuximab vedotin with chemotherapy for CD30-positive peripheral T-cell lymphoma
Source: Ann Oncol. Author manuscript; Available in PMC 2022 Sep 6. (PMC9447792; doi:10.1016/j.annonc.2021.12.002)
Supplement: 4 [file NIHMS1829921-supplement-4.docx]

**Supplementary Table S2: Summary of use of SCT by treatment group**

| **Regimen category n (%)** | **A+CHP *N*=226** | **CHOP *N*=226** | **Total  *N*=452** |
| --- | --- | --- | --- |
| Overall use of SCT | 68 (30) | 64 (28) | 132 (29) |
| Autologous | 63 (28) | 52 (23) | 115 (25) |
| Allogeneic, related donor | 3 (1) | 6 (3) | 9 (2) |
| Allogeneic, unrelated donor | 9 (4) | 13 (6) | 22 (5) |
| Use of SCT as consolidation | 50 (22) | 39 (17) | 89 (20) |
| Autologous | 49 (22) | 39 (17) | 88 (19) |
| Allogeneic, unrelated donor | 1(0) | 0 | 1 (0) |
| Use of SCT as subsequent therapy | 23 (10) | 31 (14) | 54 (12) |
| Autologous | 14 (6) | 13 (6) | 27 (6) |
| Allogeneic, related donor | 3 (1) | 6 (3) | 9 (2) |
| Allogeneic, unrelated donor | 8 (4) | 13 (6) | 21 (5) |

A+CHP, brentuximab vedotin, cyclophosphamide, doxorubicin, and prednisone; CHOP*,* cyclophosphamide, doxorubicin, vincristine, and prednisone; SCT, stem-cell transplantation
