# Supplementary material for: The ECHELON-2 Trial: 5-year results of a randomized, phase III study of brentuximab vedotin with chemotherapy for CD30-positive peripheral T-cell lymphoma
Source: Ann Oncol. Author manuscript; Available in PMC 2022 Sep 6. (PMC9447792; doi:10.1016/j.annonc.2021.12.002)
Supplement: 5 [file NIHMS1829921-supplement-5.docx]

**Supplementary Table S3: Summary of subsequent anti-cancer treatments by treatment group**

| **Regimen category n (%)** | **A+CHP *N*=226** | **CHOP *N*=226** | **Total  *N*=452** |
| --- | --- | --- | --- |
| BV monotherapy ^a^ | 24 (11) | 47 (21) | 71 (16) |
| Platinum-based chemotherapy | 21 (9) | 42 (19) | 63 (14) |
| Stem-cell transplantation | 23 (10) | 31 (14) | 54 (12) |
| Radiation | 13 (6) | 11 (5) | 24 (5) |
| Combination chemotherapy | 12 (5) | 26 (12) | 38 (8) |
| Romidepsin | 12 (5) | 10 (4) | 22 (5) |
| Single-agent or unspecified chemotherapy | 12 (5) | 17 (8) | 29 (6) |
| Other (investigational) | 8 (4) | 7 (3) | 15 (3) |
| Other (miscellaneous) | 7 (4) | 1 (0) | 8 (2) |
| BV + chemotherapy ^a^ | 5 (2) | 8 (4) | 13 (3) |
| Rituximab + chemotherapy | 5 (2) | 2 (1) | 7 (2) |
| Methotrexate | 4 (2) | 8 (4) | 12 (3) |
| Rituximab | 4 (2) | 1 (0) | 5 (1) |
| Bendamustine | 3 (1) | 5 (2) | 8 (2) |
| Pralatrexate | 3 (1) | 7 (3) | 10 (2) |
| Romidepsin-containing regimen | 3 (1) | 6 (3) | 9 (2) |
| Belinostat-containing regimen | 2 (1) | 2 (1) | 4 (1) |
| Gemcitabine monotherapy | 2 (1) | 6 (3) | 8 (2) |
| Anti-PD-1/PD-L1 antibody | 2 (1) | 4 (2) | 6 (1) |
| Mogamulizumab or mogamulizumab-containing regimen | 2 (1) | 4 (2) | 6 (1) |
| BV + nivolumab | 1 (0) | 0 | 1 (0) |
| Lenalidomide | 1 (0) | 3 (1) | 4 (1) |

A+CHP, brentuximab vedotin, cyclophosphamide, doxorubicin, and prednisone; BV, brentuximab vedotin; CHOP, cyclophosphamide, doxorubicin, vincristine, and prednisone

1. Numbers are reported by regimen. One patient is reported as having received BV monotherapy and BV + chemotherapy and is included in both categories.

Subsequent anticancer therapies that patients received after frontline medication are included, sorted based on descending frequency in the A+CHP column.
